# Treatment patterns among patients with malignant pleural mesothelioma: An Italian, population‐based nationwide study

**DOI:** 10.1111/1759-7714.13456

**Published:** 2020-05-04

**Authors:** Annalisa Trama, Claudia Proto, Diego Signorelli, Marina C. Garassino, Giuseppe Lo Russo, Monica Ganzinelli, Arsela Prelaj, Carolina Mensi, Manuela Gangemi, Valerio Gennaro, Elisabetta Chellini, Adele Caldarella, Italo F. Angelillo, Valeria Ascoli, Cristiana Pascucci, Giovanna Tagliabue, Rosanna Cusimano, Francesca Bella, Fabio Falcini, Enzo Merler, Giuseppe Masanotti, Antonio Ziino, Maria Michiara, Gemma Gola, Cinzia Storchi, Lucia Mangone, Maria F. Vitale, Claudia Cirilli, Rosario Tumino, Tiziana Scuderi, Anna C. Fanetti, Silvano Piffer, Marcello Tiseo, Gemma Gatta, Laura Botta, the LUME study WG

**Affiliations:** ^1^ Research Department Fondazione IRCCS Istituto Nazionale dei Tumori Milan Italy; ^2^ Department of Medical Oncology Fondazione IRCCS, Istituto Nazionale dei Tumori Milan Italy; ^3^ COR Lombardy, Epidemiology Unit,Fondazione IRCCS Ca' Granda Ospedale Maggiore Policlinico and University of Milan Milan Italy; ^4^ Unit of Cancer Epidemiology University of Turin and CPO‐Piemonte Turin Italy; ^5^ COR Liguria, UO Epidemiology IRCCS Ospedale Policlinico San Martino Genoa Italy; ^6^ Unit of Occupational & Environmental Epidemiology, Tuscan Occupational Cancer Registry Institute for Cancer Research, Prevention and Clinical Network (ISPRO) Florence Italy; ^7^ Tuscan Cancer Registry, Institute for Cancer Research Prevention and Clinical Network (ISPRO) Florence Italy; ^8^ Department of Experimental Medicine, COR Campania University of Campania "Luigi Vanvitelli" Naples Italy; ^9^ Department of Radiological Sciences, Oncology and Anatomical Pathology, COR Lazio University La Sapienza Rome Italy; ^10^ Mesothelioma Marche Registry ‐ COR Marche, School of Medicinal and Health Products Sciences University of Camerino Camerino Italy; ^11^ Varese Cancer Registry Fondazione IRCCS Istituto Nazionale dei Tumori Milan Italy; ^12^ Palermo Cancer Registry Palermo Italy; ^13^ Integrated Cancer Registry of Catania‐Messina‐Siracusa‐Enna Azienda Ospedaliero‐Universitaria Policlinico‐Vittorio Emanuale Catania Italy; ^14^ Romagna Cancer Registry, Istituto Scientifico Romagnolo per lo Studio e la Cura dei Tumori (IRST) IRCCS Forlì Italy; ^15^ COR Veneto, Occupational Health Unit, Department of Prevention Padua Italy; ^16^ Sec. Public Health, Department of Experimental Medicine, COR Umbria University of Perugia Perugia Italy; ^17^ Siracusa Cancer Registry Siracusa Italy; ^18^ Parma Cancer Registry Parma Italy; ^19^ Como Cancer Registry Como Italy; ^20^ Servizio di Epidemiologia Azienda Unità Sanitaria Locale – IRCCS di Reggio Emilia Reggio Emilia Italy; ^21^ U.O.S.D. Napoli 3 South Cancer Registry Piazza San Giovanni Naples Italy; ^22^ Modena Cancer Registry Modena Italy; ^23^ Cancer Registry and Histopathology Deaprtment, COR Sicily, 'Civic ‐M.P. Arezzo' Hospital Ragusa Italy; ^24^ Trapani Cancer Registry Trapani Italy; ^25^ Sondrio Cancer Registry Sondrio Italy; ^26^ Trento Cancer Registry, Servizio Epidemiologia Clinica e Valutativa, Azienda Provinciale per i Servizi Sanitari Trento Italy; ^27^ Department of Medicine and Surgery University of Parma Parma Italy

**Keywords:** Logistic models, mesothelioma malignant, registries, therapeutics

## Abstract

**Background:**

Malignant pleural mesothelioma (MPM) is a rare cancer with a poor prognosis. Centralization of rare cancer in dedicated centers is recommended to ensure expertise, multidisciplinarity and access to innovation. In Italy, expert centers for MPM have not been identified in all regions. We aimed to describe the treatment patterns among MPM patients across different Italian regions and to identify factors associated with the treatment patterns across the regions.

**Methods:**

We performed an observational study on a random sample of 2026 MPM patients diagnosed in 2003–2008. We included 26 population‐based registries covering 70% of the Italian population. To identify factors associated with treatment patterns, across the different regions, we fitted a multinomial logistic regression model adjusted by age, sex, stage, histology and hospital with thoracic surgical department.

**Results:**

MPM patients mostly received chemotherapy alone (41%) or no cancer‐directed therapy (36%) especially the older patients. The first course of treatment for MPM patients differed across regions. Patients from Piedmont, Liguria and Campania were more likely to receive no cancer‐directed therapy; those living in Tuscany and Sicily were more likely to get surgery; patients from Marche and Lazio were more likely to receive chemotherapy. These differences were not explained by age, sex, stage, histology and availability of a thoracic surgery department.

**Conclusions:**

There is limited expertise available and lack of a network able to maximize the expertise available may contribute to explaining the results of our study. Our findings support the need to ensure the appropriate care of all MPM patients in reorganizing the health care services.

**Key points:**

**Significant findings of the study:**

MPM patients mostly received chemotherapy alone or no cancer‐directed therapy especially the older patients. The first course of treatment for MPM patients differed across Italian regions.

**What this study adds:**

Differences in MPM clinical management are not explained by the age, stage, histology nor by the availability of a thoracic surgery department. Limited expertise for MPM contribute to explaining the unequal access to appropriate care for MPM patients in Italy.

## Introduction

Malignant pleural mesothelioma (MPM) is a rare tumour^1^ strongly associated with asbestos exposure. In Europe, the MPM incidence rates (IR) are expected to peak around 2020 in some countries, but a decrease may have already begun in others^2^, ^3^ as a consequence of legislative restrictions implemented in the 1980s. In Italy, a downturn in the occurrence of MPM is expected to occur after 2019.^4^ However, 1450 new MPM (IR 3.3 and 0.9 in males and females, respectively) patients were diagnosed in 2014 and the IR across Italian geographical areas ranged from <4/100 000 to >100/100 000.^5^

MPM has a poor prognosis (five‐year survival 9%)^6^ and no survival progresses have been observed at population level during the last decades.^6^ Traditionally, the centralization of rare cancer in dedicated centers has been recommended to ensure expertise, multidisciplinarity and access to innovation.^7^ Nevertheless, this process requires health migration, rationing of resources and a potential failure in routine care since the limited expert resources may be overwhelmed, determining waiting lists.^7^ By ensuring appropriate care of all patients regardless of the point of access, networking seems to be the most appropriate answer to rare cancers such as MPM.^7^ In Italy, specialized centers for MPM patients have not been identified in all regions and, up to 2017, only an informal professional network on rare cancers, focused mainly on sarcoma tumors, was created to provide second opinion and clinical advice on rare cancers.^8^

Against this background, we aimed to: (i) Describe the treatment patterns among MPM patients in Italy and across Italian regions; and (ii) identify patients and health care system factors associated with treatment patterns across Italian regions.

## Methods

**Figure 1:**
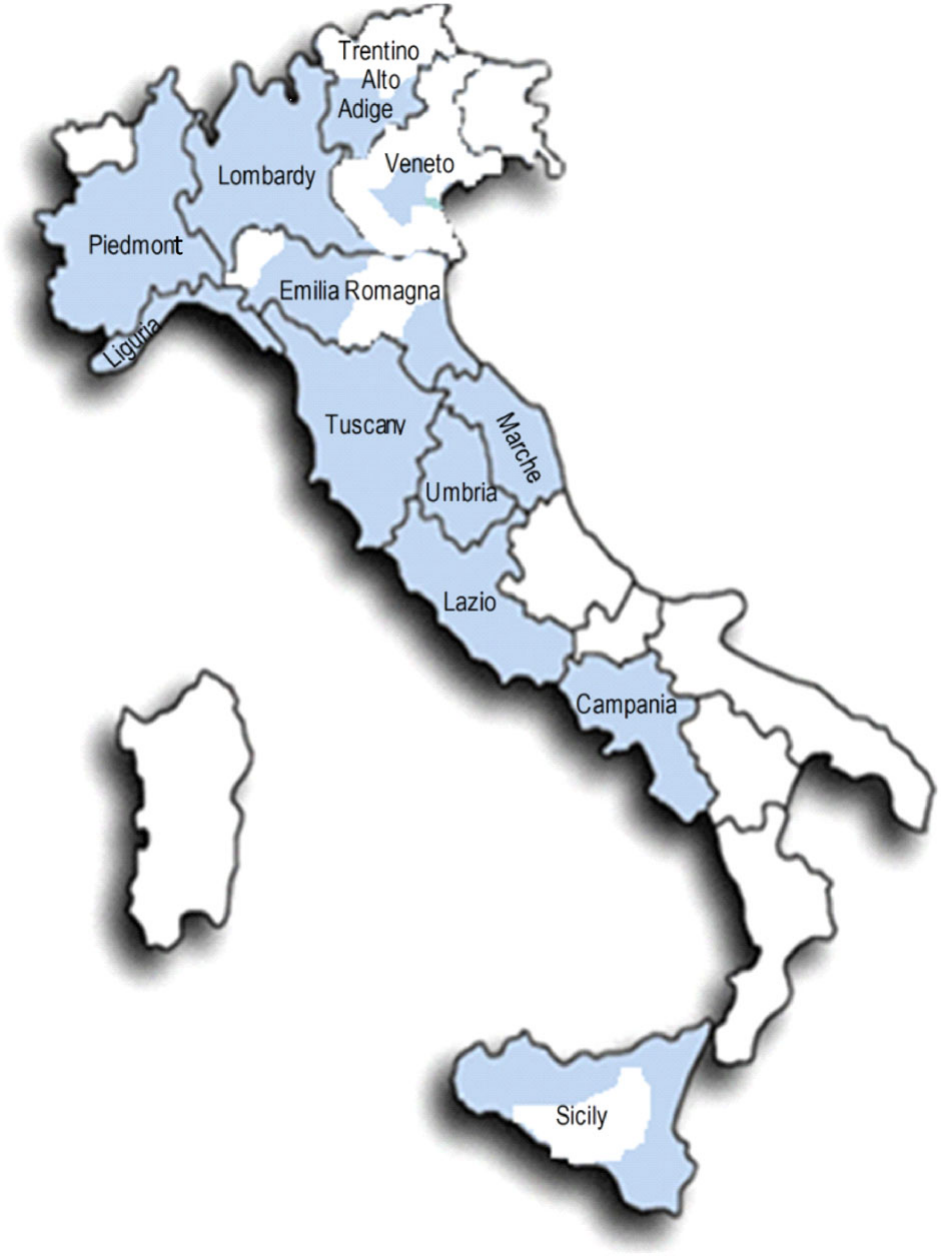
Italian geographical areas (regions) included in the study.

This population‐based study is part of the wider “MPM survivors in Italy: what is contributing to long term survival?” (LUME) project. The LUME project collaborated with 26 population‐based registries to develop a national, population‐based database of MPM patients with demographic and clinical information. Regional registries (centri operativi regionali‐COR) from the National mesothelioma‐dedicated surveillance system and general cancer registries (CRs) contributed to the LUME database. General CRs were used when a COR either was not available in a region, or did not accept a request to join the study. The 26 registries involved covered 70% of the Italian population (Fig 1) and registered 80% of all MPM cases in Italy. Veneto COR contributed with the Padua province data only; however, we will refer to it as Veneto region; Trento CR will be referred to as Trentino Alto Adige region; Siracusa, Trapani, Palermo, Catania‐Messina and Ragusa CRs will be referred to as Sicily region and Parma, Reggio Emilia, Modena and Romagna CRs as Emilia‐Romagna region (Fig 1).

About 5600 MPM, histologically or cytologically confirmed, were identified from 2003 to 2008 by the registries involved in the LUME project. Patients diagnosed from death certificate or autopsy were excluded. Clinical information are not routinely collected by these registries thus an ad hoc data collection was organized to retrieve them including diagnostic procedures, histology, clinical and pathological stage, treatment information (surgery [SRG], radiotherapy [RT], chemotherapy [CHT] and best supportive care [BSC]), hospital of origin (including information on the availability of an onsite thoracic surgical department), and follow‐up. Data collection was based on a common protocol, agreed by a multidisciplinary group including registrars, epidemiologist and MPM clinical experts (pathologists, surgeons and oncologists).

Due to restricted financial resources the LUME project could not collect data for all the 5600 cases but for a representative random sample of 2026 MPM patients, taking into consideration the relative numerical contribution of each registry.

Clinical and pathological T, N and M were those defined by the clinicians. The TNM Staging System proposed from the International Mesothelioma Interest Group was used for the analyses.^9^

We defined the first course of treatment as the one started within five months from the diagnosis:

- Surgery alone (including extra pleural pneumonectomy [EPP], pleurectomy with decortications [P/D] and pleurectomy);
- Chemotherapy alone;
- Chemotherapy and surgery (SRG + CHT);
- Multimodal approach consisting of EPP + radical RT + CHT;
- Other treatment combinations (including SRG + radical RT; CHT + radical RT; surgery≠EPP + radical RT + CHT);
- No treatment (including also BSC);
- Missing information.

For the treatment time frame please refer to Supplementary material B, Table S1.

**Table 1:** Demographic and clinical characteristics of malignant pleural mesothelioma patients diagnosed 2003–2008 in the LUME study

Variable	Category	No. of cases	%
Total		2026	100
Age class	15–54	188	9.3
	55–64	475	23.4
	65–74	733	36.2
	75+	630	31.1
Sex	Male	1438	71
	Female	588	29
Histotype	Epitheliod	1384	68.3
	Not otherwise specified	236	11.7
	Biphasic	229	11.3
	Sarcomatoid	177	8.7
Diagnostic confirmation	Histological	1917	94.6
	Cytological	109	5.4
Imaging	CT scan or/and PET or/and MRI	1818	89.7
	X‐ray alone	164	8.1
	None	44	2.2
Clinical stage**†**	Stage I–II	928	45.8
	Stage III	375	18.5
	Stage IV	426	21.0
	Missing information	297	14.7
First course of treatment	Surgery alone	135	6.7
	Surgery and chemotherapy	206	10.2
	Chemotherapy alone	833	41.1
	Multimodal treatment	17	0.8
	Other combination of treatments	21	1
	No treatment or best supportive care	739	36.5
	Missing information	75	3.7
Information on the type of surgery (over 341 surgery ± chemotherapy treated patients)	Extra pleural pneumonectomy	134	39.3
	Pleurectomy with decortication (P/D)	101	29.6
	Pleurectomy	82	24.1
	Information on type of surgery missing	24	7

† AJCC.^9^

We used standard age groups: 15–54; 55–64; 65–74 and 75+.^10^

To identify factors associated with patterns of treatment across the different Italian geographical areas, we fitted a multinomial logistic regression model, considering as the dependent variable the most common treatment options (CHT or SRG alone, SRG + CHT, no active treatment) with “CHT alone” acting as reference. We included as the independent variables patients/tumors characteristics available in the LUME database (age, sex, stage and histology), Italian regions and health care system factors possibly associated with receipt of MPM treatment (ie, availability of a thoracic surgery department in the hospitals). For the independent variables, the category with highest frequency was the reference (ref). The output of this model is the relative risk ratio (RRR) of those who received a specific treatment versus “CHT alone”. Two‐sided *P*‐values<0.05 were considered significant.

All statistical analyses were performed using Stata, release 13.0 (Stata Corporation, College Station, TX, USA).

## Results

Clinical and demographic patient's characteristics are summarized in Table 1. Of 2026 MPM patients, 67% were diagnosed with cancer at 65 years or more (average age: 69 years), 71% were males and 68% with epitheliod histotype. Around half of patients (46%) were diagnosed with stage I–II of disease and 41% were treated with CHT alone. EPP, P/D and pleurectomy were used in 40%, 30% and 24% of the MPM surgically treated, respectively.

A description of the first course of treatment by stage and age groups is reported in Table 2. Regardless stage, CHT was the most common treatment option at all ages, apart from elderly patients (75+ years) who received less active treatments and more BSC compare to younger patients. MPM patients with stage I–II and III were more likely to be treated with SRG as compared to those diagnosed with stage IV. The type of SRG (EPP, P/D or pleurectomy) did not differ much across the stages. Younger patients (<65 years) were most likely to receive the EPP. SRG+/‐CHT use decreased with increasing age. Combined and multimodal approaches were used more for younger patients (<65 years).

**Table 2:** First course of treatment for malignant pleural mesothelioma patients included in the study by stage and by age and stage

								
		First course of treatment (%)						
Overall stage	No. of cases	Surgery alone	Chemotherapy alone	Surgery and chemotherapy	Multimodal treatment	Other combination of treatments	No treatment or BSC**†**	Missing information
Stage I–II	928	8.8	39	13.3	1.3	0.7	33.6	3.3
Stage III	375	6.8	50.1	7.2	0.5	0.5	31.7	3.2
Stage IV	426	3.8	47.2	8.5	0.2	2.8	35.5	2
Missing	297	3.7	29	6.4	0.7	0.7	52.2	7.3
Overall	2026	6.6	41.3	10.1	0.8	1.1	36.4	3.7
15–54 years old								
Stage I–II	79	13.9	38.0	30.4	2.5	0.0	10.1	5.1
Stage III	47	6.4	51.1	6.4	2.1	2.1	27.7	4.2
Stage IV	42	11.9	52.4	14.3	0.0	2.4	14.3	4.7
Missing	20	15.0	40.0	15.0	0.0	0.0	20.0	10.0
Overall	188	11.7	44.7	19.1	1.6	1.1	16.5	5.3
55–64 years old								
Stage I–II	215	10.7	44.7	21.9	2.3	1.4	17.7	1.3
Stage III	88	9.1	48.9	18.2	1.1	0.0	17.1	5.6
Stage IV	109	3.7	51.4	16.5	0.0	4.6	22.0	1.8
Missing	63	4.8	42.9	11.1	0.0	0.0	31.8	9.4
Overall	475	8.0	46.7	18.5	1.3	1.7	20.4	3.4
65–74 years old								
Stage I–II	337	8.9	47.8	12.5	1.5	0.6	24.9	3.8
Stage III	141	5.7	63.1	5.7	0.0	0.0	24.1	1.4
Stage IV	161	3.1	59.6	6.2	0.6	2.5	26.1	1.9
Missing	94	2.2	37.2	9.6	2.1	2.1	40.4	6.4
Overall	733	6.1	52.0	9.4	1.1	1.1	27.0	3.3
75+ years old								
Stage I–II	297	6.1	25.3	3.4	0.0	0.3	61.3	3.6
Stage III	99	6.1	32.3	0.0	0.0	1.0	57.6	3.0
Stage IV	114	1.8	23.7	1.8	0.0	1.7	69.3	1.7
Missing	120	2.5	13.3	0.0	0.0	0.0	77.5	6.7
Overall	630	4.6	23.8	1.9	0.0	0.6	65.3	3.8

† Best supportive care.

In all regions most of MPM patients were males and with epitheliod histotype (Supplementary material B, Table S2). MPM patients' age distribution by geographical areas in our sample corresponded to the age distribution in the general population,^11^ older patients were diagnosed in the center and north west of Italy (eg, Tuscany, Liguria, Marche, Umbria) whereas in southern Italy (eg, Campania, Sicily) the patients were younger (Supplementary material B, Table S2). Patients were mainly diagnosed with stage I–II across regions with differences ranging from about 30% in Sicily and Trentino Alto Adige to 57% in Lombardy (Table 3a). Whereas patients with stage III and IV were diagnosed mostly in Trentino Alto Adige, Veneto, Umbria and Emilia‐Romagna (Table 3a). Compared to the other regions, in Tuscany and Sicily patients were more likely to be treated with SRG+/‐CHT (34% and 27%, respectively) whereas in Liguria and Piedmont patients were less likely to be treated (no treatment in 50% and 46%, respectively) (Table 3b). CHT use varied across geographical areas ranging from about 71% in Trentino Alto Adige to 28% in Campania (Table 3b). EPP was the most used type of surgery in all regions except for Campania, Tuscany, Umbria and Piedmont (data available from the corresponding author).

**Table 3:** Distribution of clinical stage (**a**) and the first course of treatment (**b**) of malignant pleural mesothelioma patients included in the study, by Italian geographical areas

					
(**a**)		Clinical stage (%)			
Italian geographical areas**†**	No. of cases	Stage I–II	Stage III–IV	Missing	
Lombardy	455	57	41	2	
Piedmont	371	50	32	18	
Tuscany	192	48	26	26	
Umbria	39	46	51	3	
Liguria	200	44	34	22	
Marche	75	44	47	9	
Emilia‐Romagna	118	42	50	9	
Veneto	37	41	51	8	
Lazio	156	39	49	12	
Campania	207	39	44	17	
Trentino‐Alto Adige	7	29	71	0	
Sicily	169	27	43	30	

					
**(b)**Italian geographical areas**†**	No. of cases	First course of treatment (%)**‡**			
		Chemotherapy alone	Surgery ± chemotherapy	No treatment or best supportive care	Missing
Trentino‐Alto Adige	7	71	14	14	0
Marche	75	59	17	20	0
Lazio	156	51	10	40	0
Veneto	37	49	16	22	11
Sicily	169	47	27	21	4
Lombardy	455	45	17	34	0
Umbria	39	44	10	41	0
Emilia‐Romagna	118	43	16	36	2
Piedmont	371	40	12	46	0
Liguria	200	36	13	50	1
Tuscany	192	32	34	30	4
Campania	207	28	11	35	25

† The ranking of the Italian geographical areas is the % of stage I–II in Table 3a and the % of chemotherapy alone in Table 3b.

‡ The sum of each row, Italian geographical areas, does not add up at 100% due to the lack of inclusion of multimodal and other combination of treatments that occurred in few cases across the regions.

The model results (Table 4) confirmed that SRG (RRR = 2.35) use was more common than CHT in young MPM patients (15–54 years) compared to 65–74 years old. In addition, old MPM patients (RRR = 5.32), those with missing information on stage (RRR = 2.33), females (RRR = 1.47) and those with not specified and sarcomatoid histotype (RRR = 1.7 and RRR = 1.8) were more likely to get BSC than CHT (Table 4). Use of SRG+/‐CHT was more common than CHT alone in MPM patients with stage I–II compared to all other stages. Finally, the availability of a thoracic surgery onsite increased the likelihood to receive SRG + CHT (RRR = 2.23).

**Table 4:** Age‐, stage‐, sex‐, histology‐, hospital with thoracic surgical department‐adjusted relative risk ratios (RRR) of first course of treatment in relation to Italian geographical areas and their corresponding 95% confidence interval (95% CI)

		First course of treatment RRR† (95% CI)		
Variable	Category	Surgery alone	Surgery and chemotherapy	No treatment or best supportive care
Age group	65–74	1 (ref)	1 (ref)	1 (ref)
	15–54	2.35* (1.32–4.2)	2.63* (1.6–4.34)	0.67 (0.42–1.07)
	55–64	1.49 (0.93–2.39)	2.35* (1.62–3.43)	0.81 (0.6–1.11)
	75+	1.66 (0.99–2.78)	0.41* (0.21–0.79)	5.32* (4.06–6.98)
Stage	Stage I–II	1 (ref)	1 (ref)	1 (ref)
	Stage III	0.61* (0.37–0.99)	0.48* (0.29–0.77)	0.77 (0.57–1.05)
	Stage IV	0.36* (0.20–0.64)	0.44* (0.28–0.68)	1.05 (0.79–1.41)
	Missing	0.48* (0.24–0.97)	0.46* (0.26–0.83)	2.33* (1.63–3.32)
Sex	Male	1 (ref)	1 (ref)	1 (ref)
	Female	1.01 (0.66–1.55)	0.75 (0.5–1.12)	1.47* (1.15–1.89)
Histology	Epitheliod	1 (ref)	1 (ref)	1 (ref)
	NOS or not available	0.6 (0.26–1.36)	0.89 (0.48–1.65)	1.7* (1.19–2.44)
	Biphasic	1.35 (0.77–2.37)	1.74* (1.09–2.78)	1.11 (0.77–1.61)
	Sarcomatoid	1.12 (0.55–2.31)	1.11 (0.59–2.1)	1.8* (1.21–2.67)
Region	Lombardy	1 (ref)	1 (ref)	1 (ref)
	Trentino‐Alto Adige	n.a	1.69 (0.16–17.41)	0.27 (0.02–3.06)
	Veneto	0.35 (0.04–2.72)	1.29 (0.43–3.87)	0.65 (0.26–1.65)
	Piedmont	0.71 (0.38–1.3)	0.67 (0.38–1.17)	1.73* (1.23–2.45)
	Liguria	1.48 (0.77–2.87)	0.55 (0.24–1.25)	1.87* (1.23–2.85)
	Tuscany	1.91 (0.99–3.67)	3.98* (2.31–6.85)	0.81 (0.50–1.31)
	Emilia‐Romagna	0.64 (0.23–1.72)	1.42 (0.71–2.87)	1.16 (0.70–1.93)
	Marche	0.32 (0.07–1.39)	1.53 (0.70–3.35)	0.3* (0.15–0.59)
	Umbria	0.78 (0.17–3.68)	0.75 (0.16–3.61)	1.23 (0.55–2.74)
	Lazio	0.68 (0.30–1.50)	0.35* (0.14–0.87)	1.38 (0.88–2.15)
	Campania	1.19 (0.56–2.53)	0.73 (0.34–1.55)	2.66* (1.68–4.22)
	Sicily	1.14 (0.55–2.36)	2.19* (1.24–3.87)	0.44* (0.26–0.75)
Thoracic surgery department	Yes	1.58 (0.98–2.56)	2.23* (1.43–3.5)	0.81 (0.63–1.04)

NOS, not otherwise specified, n.a., not applicable; ref, reference.

* Statistically significant.

† RRRs calculated by multinomial logistic regression modeling taking “chemotherapy alone” as reference.

The model results showed that considering system level characteristics (ie, availability of a thoracic surgical department) did not fully explain the differences on first course of treatment observed across the Italian regions. Thus, compared to MPM patients living in Lombardy and getting CHT alone, after adjusting for age, sex, histology, stage and thoracic surgery onsite MPM patients living in:

- Piedmont, Liguria and Campania had a higher RRR to be untreated,
- Tuscany and Sicily had a higher RRR to get treatment including SRG,
- Marche and Sicily had a lower RRR to be untreated and,
- Lazio had a lower RRR to get SRG + CHT (than so more likely to receive CHT alone).

No major differences were confirmed across the other geographical areas.

## Discussion

This is the first population‐based study to provide a description of the treatment patterns up to 2008 for MPM patients in Italy and across different Italian geographical areas. It is the results of a unique collaborative effort including 26 registries from 12 of 21 Italian regions corresponding to 70% of the Italian population. Previous Italian studies analyzed survival of MPM patients with limited information on treatment^10^, ^12^ and generally were focused on one region.^13^, ^14^

In our study, 36% MPM patients did not receive active cancer treatment, especially elderly patients (65%) independently of disease stage. However, in our database we had not got the information to distinguish MPM patients unsuitable for systemic therapy from those untreated because they were most likely under observation. Our results are similar to those reported in 2011 in the USA, and 29% of MPM who did not receive active cancer treatment were principally older patients.^15^

Therapeutic decisions in the elderly with cancer should not be based just on chronological age but should also take into account the patient preferences, functional age, presence of comorbidities and estimated benefits and risks.^16^, ^17^

In our study, 41% received chemotherapy alone. MPM patients were also reported to be mainly treated with CHT in Belgium (60%), Netherlands (41%) and England (37%).^18^ In Slovenia, the number of patients treated with CHT increased from 32% in 1999–2003 to 80% in 2004–2008 due to the systematic introduction of CHT.^19^ Also, in the USA in 2011, MPM patients receiving systemic therapy were 60%.^15^

In our study, 19% received a treatment including SRG. In Europe, surgery was used in 27%, 10% and 5% MPM patients in Belgium, England and Netherlands, respectively (years of diagnosis 2006–2011).^18^ In Slovenia, the number of patients treated by surgery decreased from 21% in 1999–2003 to 7% in 2004–2008.^19^ In the USA, cancer‐directed surgery was reported in 22%, 23% and 27% in MPM patients in the years 1990–2004,^20^ 1973–2009^21^ and 2011,^15^ respectively.

The available data showed a heterogeneity of treatment across geographical areas and time periods. However, CHT was confirmed as the main treatment option for MPM patients in most EU countries and USA while the multimodal approach had a limited use worldwide. These reports pre‐date the results of the MARS study^22^ and enhance the support that patients who are candidates for a multimodal approach should be included in clinical trials at highly specialized centers. In the last decade, no developments have been observed regarding systemic treatment. Cisplatin and pemetrexed have remained the standard of care in MPM patients for around 20 years. Recently, various studies have explored the role of immunotherapy and its combination with standard CHT in advanced MPM patients and preliminary results seems to predict a better survival rate compared to CHT alone. Nevertheless, it appears that CHT is the best treatment option.^23^, ^24^, ^25^

Our results showed that the majority of patients are diagnosed with stage I–II and 21% with stage IV. In the USA, two studies^20^, ^21^ reported that only 11% MPM patients are diagnosed with early disease and patients diagnosed with distant disease range from 16%^20^ to 59%.^21^ Stage definition and study period could explain the differences in stage distribution across these studies. Another study in the USA^26^ reported 28% were stage I and II; 14% stage III, 29% stage IV with 29% of stage information missing from 2005 to 2009. In our study, missing information was around 15%; 90% of cases were staged based on CT scan/RMI/PET. In any case, we cannot rule out a possible stage misclassification also in our data considering the interpersonal variability of the radiologists.^27^

In our study, we found that the first course of treatment for MPM patients differed across geographical areas. The observed differences could be due to the different availability of a thoracic surgery department but, as showed by the proposed model, these factors did not fully explain the observed differences on treatment approach across regions. We believe that these differences may be due to the limited expertise available for a rare cancer such as MPM and also to the lack of a network able to maximize the available expertise.

In Italy, three consensus conferences on the management of MPM took place in 2011, 2013 and 2015^25^, ^28^, ^29^ to develop recommendations on MPM management for public health institutions, clinicians and patients. From the health care organization, some regions (eg, Emilia‐Romagna) have identified a clinical network for the management of MPM patients. More importantly, the Italian rare cancers network was established by a formal agreement between the Ministry of Health and the different Italian Regions (“Intesa Stato‐Regioni”) in September 2017.

The time of our analyses (patients diagnosed in 2003–2008) pre‐dates implementation of regional and national initiatives to ameliorate the quality of care for MPM patients and thus provides important baseline data to evaluate such initiatives. Limitations of our study include the lack of information on comorbidity and performance status, which is relevant for interpreting the treatment choice. Strengths are the centralization of data quality checks and analyses along with the population‐based nature of this effort.

In the future, population‐based data will be crucial to assess whether changes in management policies have the desired effect to ensure the best care for all MPM patients.

## Disclosure

All authors declare no competing interests.

## Supporting information


**Appendix S1.** LUME Study Working Group.

**Table S1.** Definition of first course of treatment collected in the LUME study.
**Table S2.** Demographic characteristics and histotype of malignant pleural mesothelioma patients included in the LUME study by Italian geographical areas.
