# Supplementary material for: Treatment patterns among patients with malignant pleural mesothelioma: An Italian, population‐based nationwide study
Source: Thorac Cancer. 2020 May 4;11(6):1661–9. doi: 10.1111/1759-7714.13456 (PMC7262944; doi:10.1111/1759-7714.13456)
Supplement: Supplementary file 1 — Appendix S1. LUME Study Working Group. [file TCA-11-1661-s001.docx]

**Supplementary material A**

**LUME Study Working Group**

Barbara Dallari^1^, Dario Consonni^1^,Dario Mirabelli^2^, Carol Brentisci^2^, Lucia Benfatto^3^, Cecilia Francesca Lando^3^, Francesca Battisti^4^, Daniela Feola^5^, Francesco Napolitano^5^, Francesco Forastiere^6^, Elisa Romeo^6^, Iolanda Grappasonni^7^ , Laura Di Grazia^8^, Frassoldi Emanuela^8^, Rosalba Amodio^9^, Salvatore Sciacca^10^, Marine Castaing^10^, Orietta Giuliani^11^, Rosa Vattiato^11^, Vittoria Bressan^12^, Chiara Panato^13^, Fabrizio Stracci^14^, Francesco Tisano^15^, Anita Rimanti^16^, Paolo Sgargi^16^, Mariangela Corti ^17^, Antonio Romanelli^18^,Roberto Piro^19^, Mario Fusco^20^, Graziella Frasca^21^, Maria Concetta Giurdanella^21^ , Giuseppa Candela^22^, Roberto Vito Rizzello^23^, Ivan Cometti^24^, Alessandro Marinaccio^25^

1COR Lombardy, Epidemiology Unit, Fondazione IRCCS Ca' Granda, Ospedale Maggiore Policlinico and University of Milan, Milan, Italy.

2 COR Unit of Cancer Epidemilogy, University of Turin and CPO-Piemonte, Torino, Italy.

3COR Liguria, UO Epidemiology, IRCCS Ospedale Policlinico San Martino, Genova, Italy

4 Unit of Occupational & Environmental Epidemiology, Tuscan Occupational Cancer Registry, Institute for Cancer Research, Prevention and Clinical Network (ISPRO) Florence, Italy

5 Department of Experimental Medicine, COR Campania, University of Campania "Luigi Vanvitelli", Naples, Italy

6 COR Lazio, Dipartimento di Epidemiologia del Servizio Sanitario Regionale, Rome, Italy

7Environmental and Health Sciences Department, COR Marche, University of Camerino, Hygiene, Camerino, Italy.

8Varese Cancer Registry, Varese, Italy

9 Palermo Cancer Registry Palermo, Italy

10Integrated Cancer Registry of Catania-Messina-Siracusa-Enna, Azienda Ospedaliero-Universitaria Policlinico Vittorio Emanuele, Catania

11 Romagna Cancer Registry, Istituto Scientifico Romagnolo per lo Studio e la Cura dei Tumori (IRST) IRCCS, Meldola (FC), Italy

12COR Veneto, Occupational Health Unit, Department of Prevention, Padua, Italy.

13 Cancer Epidemiology Unit, Centro di Riferimento Oncologico di Aviano (CRO), IRCCS, Aviano , Italy;

14 Sec. Public Health, Department of Experimental Medicine, COR Umbria, University of Perugia, Perugia, Italy.

15Siracusa Cancer Registry, Siracusa, Italy

16Parma Cancer Registry, Parma, Italy

17Como Cancer Registry, Como, Italy

18Azienda USL-IRCCS di Reggio Emilia

19U.O. Pneumologia ASMN IRCCS, Reggio Emilia, Italy;

20 U.O.S.D. Napoli 3 South Cancer Registry, Brusciano (Na), Italy

21 Cancer Registry and Histopathology Deaprtment, COR Sicily, 'Civic -M.P. Arezzo' Hospital, ASP Ragusa, Italy

22Trapani Cancer Registry, Trapani,Italy

23Trento Cancer Registry, Servizio Epidemiologia Clinica e Valutativa, Azienda Provinciale per i Servizi Sanitari, Trento, Italy.

24 Sondrio Cancer Registry, Sondrio, Italy

25 Unit of Epidemiology, Department of Occupational Medicine, Italian National Institute for Occupational Safety andPrevention (ISPESL), Rome, Italy
