# Supplementary material for: Treatment patterns among patients with malignant pleural mesothelioma: An Italian, population‐based nationwide study
Source: Thorac Cancer. 2020 May 4;11(6):1661–9. doi: 10.1111/1759-7714.13456 (PMC7262944; doi:10.1111/1759-7714.13456)
Supplement: Supplementary file 2 — Table S1. Definition of first course of treatment collected in the LUME study. Table S2. Demographic characteristics and histotype of malignant pleural mesothelioma patients included in the LUME study by Italian geographical areas. [file TCA-11-1661-s002.docx]

| **Supplementary material B**  **Table 1. Definition of first course of treatment collected in the LUME study** | | | | | |  |
| --- | --- | --- | --- | --- | --- | --- |
| First course of treatment definition | Time since diagnosis | First treatment | Time from 1^th^ to 2^nd^ treatment | Second treatment | Time from 2^nd^ to 3^rd^ treatment | Third treatment |
| Surgery alone | <=5 months | Surgery* |  |  |  |  |
| Chemotherapy alone | <=5 months | Chemotherapy |  |  |  |  |
| Chemotherapy and surgery | <=5 months | Surgery* | <=4 months | Chemotherapy |  |  |
| Chemotherapy and surgery | <=5 months | Chemotherapy | <=9 months | Surgery* |  |  |
| Multimodal treatment | <=5 months | EPP | <=4 months | Chemotherapy | <=9 months | Radical Radiotherapy |
| Multimodal treatment | <=5 months | EPP | <=4 months | Radical Radiotherapy | <=4 months | Chemotherapy |
| Multimodal treatment | <=5 months | Chemotherapy | <=9 months | EPP | <=4 months | Radical Radiotherapy |
| Multimodal treatment | <=5 months | Chemotherapy | <=9 months | Radical Radiotherapy | <=4 months | EPP |
| Multimodal treatment | <=5 months | Radical Radiotherapy | <=4 months | EPP | <=4 months | Chemotherapy |
| Multimodal treatment | <=5 months | Radical Radiotherapy | <=4 months | Chemotherapy | <=9 months | EPP |
| Other combination of treatments | <=5 months | Surgery* | <=4 months | Radical Radiotherapy |  |  |
| Other combination of treatments | <=5 months | Radical Radiotherapy | <=4 months | Surgery* |  |  |
| Other combination of treatments | <=5 months | Chemotherapy | <=9 months | Radical Radiotherapy |  |  |
| Other combination of treatments | <=5 months | Radical Radiotherapy | <=4 months | Chemotherapy |  |  |
| Other combination of treatments | <=5 months | Radical Radiotherapy |  |  |  |  |
| Other combination of treatments | <=5 months | P/D or Pleurectomy | <=4 months | Chemotherapy | <=9 months | Radical Radiotherapy |
| Other combination of treatments | <=5 months | P/D or Pleurectomy | <=4 months | Radical Radiotherapy | <=4 months | Chemotherapy |
| Other combination of treatments | <=5 months | Chemotherapy | <=9 months | P/D or Pleurectomy | <=4 months | Radiotherapy |
| Other combination of treatments | <=5 months | Chemotherapy | <=9 months | Radical Radiotherapy | <=4 months | P/D or Pleurectomy |
| Other combination of treatments | <=5 months | Radical Radiotherapy | <=4 months | P/D or Pleurectomy | <=4 months | Chemotherapy |
| Other combination of treatments | <=5 months | Radical Radiotherapy | <=4 months | Chemotherapy | <=9 months | P/D or Pleurectomy |
| Not treated or Best supportive care | <=5 months | None/ Best supportive care | |  |  |  |
| Missing | Info on date unavailable, info on treatment unavailable | | | | | |
|  | *extra pleural pneumonectomy (EPP) or pleurectomy with decortications (P/D) or pleurectomy | | | | | |

**Table 2.** **Demographic characteristics and histotype of malignant pleural mesothelioma patients included in the LUME study by Italian geographical areas**

|  | | **Total** | **Italian geographical areas** | | | | | | | | | | | |
| --- | --- | --- | --- | --- | --- | --- | --- | --- | --- | --- | --- | --- | --- | --- |
|  |  |  | **Lombardy** | **Trentino-Alto Adige** | **Veneto** | **Piedmont** | **Liguria** | **Tuscany** | **Emilia-Romagna** | **Marche** | **Umbria** | **Lazio** | **Campania** | **Sicily** |
| **No. of cases** | | **2026** | **455** | **7** | **37** | **371** | **200** | **192** | **118** | **75** | **39** | **156** | **207** | **169** |
| **Age class (%)** | 15-54 | 9.3 | 7.0 | 14.3 | 8.1 | 8.4 | 5.0 | 7.8 | 6.8 | 9.3 | 15.4 | 11.5 | 17.9 | 11.8 |
|  | 55-64 | 23.4 | 24.0 | 14.3 | 24.3 | 23.1 | 21.0 | 21.4 | 28.0 | 21.4 | 18.0 | 19.9 | 25.1 | 28.4 |
|  | 65-74 | 36.2 | 36.9 | 57.1 | 43.2 | 37.5 | 34.5 | 34.9 | 34.8 | 33.3 | 28.2 | 41.7 | 35.3 | 32.5 |
|  | 75+ | 31.1 | 32.1 | 14.3 | 24.4 | 31.0 | 39.5 | 35.9 | 30.4 | 36.0 | 38.4 | 26.9 | 21.7 | 27.3 |
| **Sex (%)** | male | 71.0 | 67.6 | 60.9 | 71.4 | 66.0 | 82.0 | 77.6 | 74.6 | 80.0 | 82.1 | 69.2 | 74.4 | 77.5 |
|  | female | 29.0 | 32.4 | 39.1 | 28.6 | 34.0 | 18.0 | 22.4 | 25.4 | 20.0 | 17.9 | 30.8 | 25.6 | 22.5 |
| **Histotype (%)** | epithelioid | 68.3 | 64.9 | 66.2 | 42.8 | 71.7 | 79.5 | 65.6 | 63.6 | 57.3 | 61.6 | 82.0 | 65.2 | 59.2 |
|  | not otherwise specified | 11.7 | 5.4 | 11.2 | 28.6 | 6.7 | 7.0 | 17.7 | 14.4 | 25.3 | 10.2 | 10.3 | 13.1 | 14.8 |
|  | bifasic | 11.3 | 10.8 | 12.5 | 14.3 | 17.0 | 4.5 | 9.9 | 10.2 | 9.4 | 15.4 | 5.8 | 10.1 | 12.4 |
|  | sarcomatoid | 8.7 | 18.9 | 10.1 | 14.3 | 4.6 | 9.0 | 6.8 | 11.8 | 8.0 | 12.8 | 1.9 | 11.6 | 13.6 |
